# Asthma diagnosis and treatment – 1010. Lung function tests in exercise-induced asthma among pediatric population

**DOI:** 10.1186/1939-4551-6-S1-P10

**Published:** 2013-04-23

**Authors:** Silvia Sanchez Garcia, Pablo Rodriguez Del Rio, Carmelo Escudero, M Dolores Ibañez

**Affiliations:** 1 Allergy Section, Hospital Infantil Universitario Niño Jesus, Madrid, Spain

## Background

EIA (Exercise-induced Asthma) can be considered a marker of severity and disease control in asthma. There are several diagnostic tools to evaluate airway hyperresponsiveness in EIA, but it has not been establish the best one.

Our objective is to explore bronchial hyperresponsiveness with different lung function measurement techniques among a pediatric population with clinical history suggestive of EIA.

## Methods

We included 23 patients who complained of respiratory symptoms (cough, chest tighness, dyspnea and/or wheezing) due to physical exercise. Lung function was monitored 5, 10, 15 and 30 min after exercise in 13 of these patients after reproducing the same exercise eliciting symptoms, with an Asthma Monitor (AMOS®, Jaeger). Exercise challenge, methacoline and manitol tests were performed in our outpatient clinic in all the 23 patients.

## Results

Patients were aged between 8 and 16 years old (mean 12, ±2.27). Twelve out of 13 (92%) patients monitoring lung function showed a decrease in lung function after exercise.

A positive methacoline challenge test was observed in 21 subjects (87,50%) (PC_20_ from 0,08 to 10,51 mg/ml (mean 0.90, ±2,93). Manitol test was positive in 16 patients (66,67%), showing a PD_15_ from 15 to 475 mg (178.71, ±186,61). Only 2 children (8%) resulted in a positive exercise challenge.

## Conclusions

According to our results, exercise challenge shows a high percentage of negative results, what might be due to difficulties reproducing the same conditions when the exercise is performed in a real life setting. Lung function monitoring resulted in a high percentage of positive results, and also provided information regarding the elapsed time between the exercise discontinuation and the maximum lung function fall recorded. However, this technique is not standardized yet. Methacoline was the test detecting the highest rate of positive results mirroring bronchial hyperresponsiveness in a pediatric population with a clinical history of EIA.

